# Isolation of *Salmonella* spp. from black spiny‐tailed iguana (*Ctenosaura similis*) meat commercialised in markets of León city, Nicaragua

**DOI:** 10.1002/vms3.654

**Published:** 2021-10-06

**Authors:** Rosmary Ríos, Byron Flores, Brenda Mora‐Sánchez, Dayana Torres, Jessica Sheleby‐Elías, William Jirón, José L. Balcázar

**Affiliations:** ^1^ Centro Veterinario de Diagnóstico e Investigación (CEVEDI) Departamento de Veterinaria y Zootecnia Escuela de Ciencias Agrarias y Veterinarias Universidad Nacional Autónoma de Nicaragua‐León (UNAN‐León) León Nicaragua; ^2^ Catalan Institute for Water Research (ICRA) Girona Spain; ^3^ University of Girona Girona Spain

**Keywords:** antimicrobials, foodborne diseases, iguana, meat hygiene, *Salmonella*

## Abstract

**Background:**

The black spiny‐tailed iguana (*Ctenosaura similis*) is an endemic animal in Mesoamerica, whose meat is consumed by the local population.

**Objectives:**

Because the black spiny‐tailed iguana may be potential reservoirs of pathogens, this study aimed to isolate and characterise *Salmonella* spp. in their meat commercialised in markets of the city of León, Nicaragua.

**Methods:**

Thirteen specimens were analysed for the isolation of *Salmonella* spp., as well as their antimicrobial resistance patterns, including the presence of genes encoding extended‐spectrum β‐lactamases.

**Results:**

*Salmonella* spp. isolates were found in eight out of 13 samples, with *S. enterica* serovar Enteritidis being found in six out of eight samples. Moreover, eight *Salmonella* spp. isolates were resistant to amoxicillin plus clavulanic acid and cephalexin, but sensitive to other tested antibiotics. The *bla*_SHV_ gene was detected in seven out of eight *Salmonella* spp. isolates, followed by the *bla*_TEM_ (two out of eight) and *bla*_CXT‐M_ (one out of eight) genes.

**Conclusions:**

These findings represent an important contribution to the implementation of appropriate strategies to prevent foodborne diseases.

## Abstract 2

A high frequency of *Salmonella* was observed in *Ctenosura similis* meat in markets of the city of León. *Salmonella enterica* serovar Enteritidis was the most frequent. Moreover, high antimicrobial resistance to amoxicillin/clavulanic acid and cephalexin was observed coinciding with the presence of the *bla_SHV_* gene.

## 1 INTRODUCTION

Nicaragua has a rich and extensive biodiversity, which is greatly influenced by its geographical position, as a bridge in the centre of the Americas that allows the movement of species from the north to the south and vice versa (Sistla et al., 2016). The wild species most exploited for human consumption have been turtles, followed by the black spiny‐tailed iguana (*Ctenosaura similis*), with the latter playing an important role in the diet of the Nicaraguan population (González‐García et al., 2009). However, these wild reptiles are in contact with different pathogens, whereby posing a health risk to consumers or handlers of this species (Ebani, 2017).

The black spiny‐tailed iguana (locally known in Spanish as ‘garrobo negro’) is an endemic animal in Mesoamerica, whose geographic distribution extends from Mexico to Panama. It has been found in both tropical dry and humid forest habitats from sea level to 800 m, becoming one of the food sources for the communities (Rosales et al., 2020). In Nicaragua, this species lives in several areas and the population consumes them due to their palatability and high protein content, thereby they are subjected to indiscriminate hunting and illegal trafficking (González‐García et al., 2009). This causes massive consumption, and therefore we can find them in different markets of Nicaragua. However, there is a high microbiological risk as they are free‐living animals that do not have health plans, despite being natural reservoirs of most pathogens, such as *Salmonella* spp. and *Escherichia coli* (Morrison & Rubin, 2020). Moreover, previous studies have demonstrated a high prevalence of extended‐spectrum β‐lactamase (ESBL)‐producing *Enterobacteriaceae* isolated from humans and farm animals in Nicaragua (Hasan et al., 2016; O'Neal et al., 2020). This is of great concern to public health because ESBLs limit the effectiveness of β‐lactam antibiotics, which are widely used to treat infections associated with Gram‐negative bacteria (Rawat & Nair, 2010). Therefore, this study aimed to isolate and characterise *Salmonella* spp. in *C. similis* meat commercialised in markets of the city of León, Nicaragua. Because of their public health implications, the presence of ESBL genes was also assessed among *Salmonella* spp. isolates.

## 2 MATERIALS AND METHODS

Thirteen black spiny‐tailed iguana carcasses (one per stall) were collected from the four existing markets (La Estación *n *= 3, La Terminal *n *= 5, Central *n *= 3 and Sutiava *n *= 2) in the city of León, Nicaragua, which has a population of 210,041 inhabitants (INIDE, 2021). The sample number and selection corresponded to the quantity of specimens for sale at the time of the sampling, which was carried out for two consecutive days.

The eviscerated carcasses that should be less than 2 h after the slaughter at the markets were stored in individual polyethylene bags duly identified and sterilised by ultraviolet radiation. Later, they were transported in a portable refrigerator at 4°C to avoid bacterial proliferation. At the time of sample collection, a file was filled out for the general characteristics of these and the way in which merchants offered the product to the population.

### 2.1 Bacteriological analysis

To isolate *Salmonella* spp., 25.0 g of carcass leg muscle was weighed, by making previously an incision with a sterile scalpel to collect the sample using sterile tweezers. Samples were then placed in a sterile bag and 225 ml of sterile buffered peptone water (OXOID, Waltham, MA, USA) was added, which was further homogenised and incubated at 35°C for 18 h. A volume of 1 ml of this pre‐enrichment phase was transferred to a tube with 10 ml of Rappaport‐Vassiliadis broth (OXOID) and incubated at 42 ± 1°C for 24 h (Donaghy & Madden, 1993). Subsequently, 100 μl of this enrichment was spread on selective agar plates, such as *Salmonella Shigella* Agar and MacConkey Agar (OXOID), which were incubated at 35°C for 24 h (Andrews et al., 2007).

Representative isolates were selected and identified based on colony morphology, Gram stain, triple sugar iron, lysine iron agar, Simmons citrate, motility‐indole‐ornithine, and confirmed with the biochemical test set of the API 20E commercial kit (bioMérieux, Marcy l'Etoile, France).

For the molecular identification of *Salmonella* spp. (389‐bp for *invA* gene), *Salmonella enterica* serovar Enteritidis (299‐bp for *sdf* gen) and *S. enterica* serovar Typhimurium (433‐bp for *fliC* gen), polymerase chain reaction (PCR) assays were applied using the primers described in Table 1. It should be noted that *S. enterica* serovars Enteritidis and Typhimurium are the main agents associated with foodborne disease (Hendriksen et al., 2011). All the amplification reactions were carried out in a final volume of 15 μl, which contained 7.5 of MasterMix 2X (Promega, USA), 1.5 μl nuclease‐free water, 5 μl genomic DNA and 0.5 μl of each specific primer at 500 nM. The amplification conditions included 94°C for 5 min, followed by 35 cycles of 94°C for 30 s, 65°C for 1 min and 72°C for 1 min, and a final extension at 72°C for 7 min.

**TABLE 1:** List of primers used for the detection of *Salmonella* and extended‐spectrum β‐lactamases (ESBLs)

Target identification	Forward	Reverse	Gene	Product (pb)	Sources
*Salmonella* spp.	5´‐GCTGCGCGCGAACGGCGAAG‐3´	5´‐TCCCGCCAGAGTTCCCATT‐3´	*invA*	389	Ferretti et al. (2001)
*S. enterica* serovar Enteritidis	5´‐AAATGTGTTTTATCTGATGCAAGAGG‐3´	5´‐GTTCGTTCTTCTGGTACTTACGATGAC‐3´	*sdf*	299	O'Regan et al. (2008)
*S. enterica* serovar Typhimurium	5´‐CCCCGCTTACAGGTCGACTAC‐3´	5´‐AGCGGGTTTTCGGTGGTTGT‐3´	*fliC*	433	O'Regan et al. (2008)
*bla*_TEM_	5´‐TCCGCTCATGAGACAATAACC‐3´	5´‐TTGGTCTGACAGTTACCAATGC‐3´	TEM	931	Kiratisin et al. (2008)
*bla*_SHV_	5´‐TGGTTATGCGTTATATTCGCC‐3´	5´‐GGTTAGCGTTGCCAGTGCT‐3´	SHV	868	Kiratisin et al. (2008)
*bla*_CTX‐M_	5´‐TCTTCCAGAATAAGGAATCCC‐3´	5´‐CCGTTTCCGCTATTACAAAC‐3´	CTX‐M	909	Kiratisin et al. (2008)

The resistance patterns were determined by the agar diffusion method, according to the protocol established by the Clinical Laboratory Standards Institute (CLSI) (Uddin et al., 2018). Briefly, a bacterial suspension was prepared at a concentration of 0.5 on the McFarland scale (5 × 10^8^ CFU/ml), which was inoculated on Mueller Hinton agar plates, and the disks impregnated with the following antibiotics were placed: tetracycline (TET), ciprofloxacin (CIP), amoxicillin/clavulanic acid (AMC), trimethoprim/sulfamethoxazole (TMP/SMX), cephalexin (CL), and gentamicin (CN). The plates were incubated at 37°C for 24 h, the inhibition halos were measured and the results were recorded as resistant (R), intermediate (I) and sensitive (S), as previously described (Carpenter et al., 2018).

For the detection of ESBLs, such as TEM, SHV and CTX‐M, the primers described in Table 1 were used. The amplification reaction was carried out in a final volume of 15 μl, which contained 7.5 of MasterMix 2X (Promega), 1.5 μl of nuclease‐free water, 0.5 μl of each specific primer at 500 nM and 5 μl of genomic DNA. The PCR amplification consisted of an initial denaturation of 94°C, followed by 40 cycles of denaturation at 94°C for 1 min, annealing at 65°C for 1 min and extension at 72°C for 1 min. The final extension was carried out at 72°C for 7 min. PCR products were visualised by agarose gel electrophoresis (2% w/v) stained with ethidium bromide.

### 2.2 Statistical analysis

The results were analysed as relative frequencies with their respective 95% confidence intervals. Fisher's exact test was applied to determine the significant association between categorical variables.

## 3 RESULTS

The frequency of *Salmonella* spp. isolated using the culture method was eight out of 13 (61.53, CI 95%: 31.57–86.14), regardless of the market. *Salmonella* spp. was isolated in the four markets with a higher proportion (three out of four) in the “La Terminal” market, whereas the frequency was two out of three in the Central and “La Estación” markets, respectively. The only sample taken from the “Sutiava” market was positive for *Salmonella* spp. No significant differences were observed in the isolation of *Salmonella* between the markets (*p *≥ 0.05).

PCR analysis demonstrated that isolates belonging to the genus *Salmonella* were found in eight out of 13 samples, with *S. enterica* serovar Enteritidis being found in six out of eight (Table 2). Moreover, the analysis of the resistance patterns showed that eight out of eight *Salmonella* isolates were resistant to AMC and CL, but sensitive to CIP, TMP/SMX, CN and TET. The *bla*_SHV_ gene was detected in seven out of eight *Salmonella* isolates, whereas the *bla*_TEM_ gene was detected in two out of eight and the *bla*_CXT_ gene in one out of eight *Salmonella* isolates. One isolate from the “La Estación” market was positive for three analysed β‐lactamase genes, whereas one isolate from the “Central” market was negative for all analysed β‐lactamase genes (Table 2).

**TABLE 2:** Identification, genotypic and phenotypic antimicrobial resistance patterns of *Salmonella* isolates from *Ctenosaura similis* meat

		*Salmonella* identification PCR			Genotypic antimicrobial resistance pattern			Phenotypic antimicrobial resistance profile					
Isolate	Markets	*Salmonella* spp.	*S. enterica* ser. Typhimurium	*S. enterica* ser. Enteritidis	*bla*_TEM_	*bla*_SHV_	*bla*_CTX‐M_	AMC	TMP/SMX	TET	CN	CL	CIP
1	Sutiava	+	–	+	–	+	–	R	S	S	S	R	S
3	Central	+	–	–	–	+	–	R	S	S	S	R	S
4	Central	+	–	+	–	–	–	R	S	S	S	R	S
5	La Estación	+	–	+	–	+	–	R	S	S	S	R	S
6	La Estación	+	–	+	+	+	+	R	S	S	S	R	S
8	La Terminal	+	–	+	+	+	–	R	S	S	S	R	S
9	La Terminal	+	–	+	–	+	–	R	S	S	S	R	S
11	La Terminal	+	–	–	–	+	–	R	S	S	S	R	S

Positive (+), negative (−), amoxicillin/clavulanic acid (AMC), trimethoprim/sulfamethoxazole (TMP/SMX), tetracycline (TET), gentamicin (CN), cephalexin (CL), ciprofloxacin (CIP).

## 4 DISCUSSION

*Salmonella* is one of the main bacteria that has been involved in outbreaks due to the consumption of contaminated food. In fact, the Centers for Disease Control and Prevention estimates that causes one million cases of food‐related illnesses each year in the USA (Callejón et al., 2015). Moreover, the poor hygiene habits of certain populations may trigger disease episodes (Waldman et al., 2020).

*Salmonella* spp. was found in 61.53% of the analysed samples, whose data are close to those obtained from previous studies. For instance, it has been reported the presence of *Salmonella* in 54.1% reptiles (e.g., turtles, lizards and snakes) from Germany and Austria (Geue & Löschner, 2002). Similar data have also been reported in Puerto Rico, where *Salmonella* spp. was detected in 52.5% of green iguana meat (Ramos et al., 2017). It is expected therefore to find a high prevalence of *Salmonella* spp. in these species, as they form part of the saprophytic microbiota (Geue & Löschner, 2002).

Although previous studies have demonstrated the occurrence of human infections with uncommon *Salmonella* serotypes (e.g., Cotham and Kisarawe) linked to reptile pets (Kiebler et al., 2020), most of them are associated with contamination of the microbiota, either skin or cloaca (Rosales et al., 2020). Moreover, most of the information on *Salmonella* spp. in reptile meat comes from crocodiles, but there are some reports of *S. enterica* serovar Chester in sea turtle meat (Magnino et al., 2009), *S. enterica* serovar Typhimurium isolated from snapping turtle meat (Fukushima et al., 2008) and *S. enterica* serovars Anatum and Baildon isolated from alligator meat (Xia et al., 2009). However, limited information is available on the presence of *Salmonella* spp. in iguana meat (Ramos et al., 2017).

In this study, *S. enterica* serovar Enteritidis was found with the highest frequency, which is one of the most frequently associated with foodborne diseases (Hendriksen et al., 2011). Two isolates of *Salmonella* spp. could not be identified at the serovar level. It is possible that they could belong to other serovars that are frequently found in reptiles, such as Rubislaw, which has recently been found in iguana and cane toads in Grenada (Sylvester et al., 2014).

Additionally, high resistance to AMC and CL was observed among *Salmonella* isolates. These findings are similar to those observed in another study in which a high frequency of *Enterobacteriaceae* resistant to penicillins and cephalosporins was observed (Amadi et al., 2015). Previous studies have also reported a lower resistance, as is the case of a study carried out in 2002 in which AMC resistance was only 31.51% in *Salmonella* isolated from domestic reptiles (Ebani et al., 2005). These data confirm that the indiscriminate use of antibiotics in humans and animals has increased the development of resistance in bacteria from other animal species, including species considered exotic or wild.

The PCR assays demonstrated that the *bla*_SHV_ gene was the most frequently detected among *Salmonella* isolates. Similar results have previously been described, in which ESBL belonging to the *bla*_TEM_ or *bla*_SHV_ families have been found in *Salmonella* isolates; however, other unrelated enzyme groups, such as *bla*_PER_ and *bla*_CTX‐M_, have also been described (Weill et al., 2004). ESBL production is a resistance mechanism of Gram‐negative bacteria and they are derived from the broad‐spectrum β‐lactamases. These enzymes confer resistance to all oxyimino‐cephalosporins, thus inactivating penicillins, monobactams and first, second, third and fourth generation cephalosporins (Del Pozo et al., 2006). Therefore, the PCR results support the high resistance towards AMC and CL observed in the phenotypic evaluation (Kirby‐Bauer method).

## 5 CONCLUSIONS

A high frequency of *Salmonella* was observed in *C. similis* meat commercialised in markets of the city of León. Among them, *S. enterica* serovar Enteritidis was the most frequently detected. Moreover, high resistance to AMC and CL was observed among *Salmonella* isolates, whose results were supported by the presence of the *bla*_SHV_ gene. To the best of our knowledge, this is the first study on the microbiological quality of the meat from this species, which may contribute to the implementation of appropriate public health strategies and mitigation programs of foodborne diseases.

## CONFLICT OF INTEREST STATEMENT

The authors declare that they have no conflict of interest.

## ETHICS STATEMENT

To carry out this study, the anonymity of the merchant and the participating establishments was preserved. The objectives of the study were also explained to each merchant and informed consent was requested, explaining the possible benefits and drawbacks. This study was previously approved by the Research Commission of the School of Agricultural and Veterinary Sciences (ECAV), Universidad Nacional Autónoma de Nicaragua, León (UNAN‐León).

## AUTHOR CONTRIBUTIONS

*Conceptualisation, data curation, formal analysis, investigation, methodology, resources, supervision, writing original draft, writing review and editing*: Rosmary Ríos. *Conceptualisation, data curation, formal analysis, investigation, methodology, supervision, writing original draft, writing review and editing*: Byron Flores. *Conceptualisation, data curation, formal analysis, writing original draft*: Brenda Mora‐Sánchez. *Data curation, formal analysis, investigation, methodology, writing original draft, writing review and editing*: Dayana Torres. *Conceptualisation, formal analysis, methodology, writing original draft, writing review and editing*: Jessica Sheleby‐Elías. *Conceptualisation, formal analysis, investigation, methodology, writing original draft*: William Jirón. *Formal analysis, methodology, supervision, writing original draft, writing review and editing*: Jose Luis Balcazar.

### PEER REVIEW

The peer review history for this article is available at https://publons.com/publon/10.1002/vms3.654

## Data Availability

All data are available on request from the authors.
